# Incidence of Augmentation in Primary Restless Legs Syndrome Patients May Not Be That High: Evidence From A Systematic Review and Meta-Analysis

**DOI:** 10.1097/MD.0000000000002504

**Published:** 2016-01-15

**Authors:** Guang Jian Liu, Lang Wu, Song Lin Wang, Li Ding, Li Li Xu, Yun Fu Wang, Li Ying Chang

**Affiliations:** From the Department of Neurology, Taihe Hospital Affiliated to Hubei University of Medicine, Shiyan City, Hubei Province, China (GJL, SLW, LD, LLX, YFW); Division of Epidemiology, Department of Medicine, Vanderbilt Epidemiology Center, Vanderbilt University School of Medicine, Nashville, TN (LW); Center for Clinical and Translational Science, Mayo Clinic, Rochester, MN (LW); and Department of Neurology, Xiangyang Center Hospital Affiliated to Hubei University of Arts and Science, Xiangyang City, Hubei Province, China (LYC).

## Abstract

Augmentation is a common complication of primary restless legs syndrome (RLS) during treatment; however, its incidence rate remains unclear.

The aim of this study is investigate the rate of augmentation during RLS treatment.

We searched 6 databases, including PubMed, OVID, Embase, Wiley citations, Web of Science research platform (including SciELO Citation Index, Medline, KCI Korean Journal Database, the Web of Science™ Core Collection), and the Cochrane library, and screened the reference lists of the included trials and recently published reviews.

Randomized controlled trials and observational studies that reported augmentation events during RLS treatment.

Primary RLS patients older than 18 years.

No restrictions regarding intervention types were applied.

Three investigators independently extracted and pooled the data to analyze the augmentation rate of the total sample and of patient subgroups with different interventions, treatment durations and drug regimens and different geographic origins. Fixed-effects or random-effects model was used for pooled analysis.

A total of 60 studies involving 11,543 participants suggested an overall augmentation rate of 5.6% (95% confidence intervals (CI), 4.0–7.7). The augmentation incidence was 6.1% (95% CI, 4.1–9.1) for long-term treatment and 3.3% (95% CI, 1.4–7.3) for short-term treatment. In addition, 27.1% (95% CI, 12.3–49.5) of the levodopa-treated patients, 6.0% (95% CI, 4.1–8.8) of the patients treated with dopamine agonists, and 0.9% (95% CI, 0.2–3.3) of the patients taking pregabalin or gabapentin developed augmentation. Augmentation occurred in 7.2% (95% CI, 5.0–10.3) of the patients taking immediate-release drugs and in 1.7% (95% CI, 0.6–5.0) of the patients taking transdermal application.

The main limitations are that the augmentation rates were not evaluated according to drug dosage, gender, and age and symptom severity.

Approximately 5 to 6 in 100 RLS patients developed augmentation during treatment.

## Abstract 2

Supplemental Digital Content is available in the text

## INTRODUCTION

Restless legs syndrome (RLS) is a disturbing disorder that is clinically characterized by unpleasant sensations in the legs that are associated with an urge to move the extremities. Because the symptoms are worsened in the evening and during rest, RLS seriously affects patients’ sleep and quality of life,^1^ which leads to significantly mental and emotional disturbances.^2^ The prevalence of RLS is 3.9% to 15% adults in general^3^ and increases with age.^4^ Although less than one-third of RLS patients require treatment,^4^ a portion of treated patients do not achieve satisfactory outcomes or require long-term medication for continued relief.^5^ However, long-term medication use can result in a loss of effectiveness, reduced tolerance and augmentation,^6^ particularly among patients using dopaminergic drugs.

Augmentation remains a widely concerned complication for RLS patients. The underlying mechanisms of augmentation remain unknown,^7^ resulting in a lack of effective interventions.^7^ More importantly, in some patients, augmentation forces changes or even the termination of treatment.^7^ Augmentation was firstly discovered in levodopa-treated patients^8^ and was later observed in patients on dopamine agonists^9^; currently, there are also reports of this complication in patients on either pregabalin^10^ or gabapentin^11^ and even patients who were given only a placebo.^11^ Some researchers believe that augmentation often occurs in patients who are receiving long-term treatment,^12^ but in fact, it is not rare during short-term treatment.^13^ The earliest reported augmentation rate was 73%^8^; in the past 2 decades, the augmentation rates reported in clinical trials have varied greatly. Some researchers have summarized the prevalence of augmentation,^12^ but they did not provide pooled data. Thus, questions remain regarding the overall augmentation rate among RLS patients and the augmentation rate in patient subgroups with different treatment durations, drug regimens, and geographic origins. To address these questions, we conducted this study.

## MATERIALS AND METHODS

This systematic review and meta-analysis were performed according to the Preferred Reporting Items for Systematic Reviews and Meta-analyses statement (PRISMA).^14^ There are no ethical issues involved in our study for our data were based on published studies.

### Search Strategies

In this study, databases of PubMed, OVID, Embase, Wiley citations, Web of Science, and the Cochrane library were searched from the earliest inception to October 21, 2014 without restriction of language. The key words included intervention, study design, and endpoint event. Detailed information regarding the search terms is provided in the Supplementary Materials. In addition, we screened the reference lists of all included trials and relevant reviews to identify additional eligible studies.

### Study Selection

The inclusion criteria were as follows: Participants: all included patients were older than 18 years and were diagnosed with primary RLS according to the International Restless Legs Syndrome Study Group diagnostic criteria^15^ or the clinical version of the Hopkins telephone diagnostic interview or other criteria specified. Pregnant women and individuals with serious chronic kidney disease or Parkinson disease were excluded. Intervention: no restrictions regarding intervention types were applied. Endpoints: the evaluated endpoints included RLS augmentation. All studies that reported augmentation or events with symptoms similar to augmentation, such as relapse, malignant RLS, reemergence, recurrence, or hyperkinesia, were included in this study. Study design: randomized controlled trials, nonrandomized controlled trials, cohort studies, case–control studies, cross-sectional studies, case series, and open-label trials were included. Selective case reports (eg, case reports that only reported patients with RLS augmentation), single-case reports, trials that did not report augmentation events (no including the trials that reported zero events), trials that reported only patients with periodic limb movements during sleep, and duplicate publications were excluded from this study.

### Endpoint Definitions

Augmentation was defined as the symptomatic worsening of RLS manifested as an earlier onset of symptoms (in the afternoon or evening), a rapid onset or shorter latency of symptoms at rest, more severe symptoms, the progression of RLS symptoms to other body parts (such as the upper extremities and trunk or face), and/or a shortened duration of medication effectiveness.^16^ Moreover, compared with the natural disease course, the symptoms tend to be more severe^10^ or to fluctuate in a contradictory manner, that is, worsening with increased medication dosages.^7^ The definitions of relapse, malignant RLS, reemergence, and hyperkinesia described by the authors of the included studies are similar to augmentation; thus, we classified all of them as “augmentation” in this study. The diagnostic criteria for augmentation were based on 2003^17^ or the Max Planck Institute's (MPI) criteria,^18^ and the scoring standards were based on the Augmentation Severity Rating Scale (ASRS).^6^

### Subgrouping

For treatment duration, we defined short-term as <6 months or 24 weeks and long-term as ≥6 months or 24 weeks. N/A refers to observational studies that lack a clear indication of treatment duration. Regarding intervention, we divided patients into subgroups according to whether they used dopamine agonists, levodopa (including levodopa, levodopa/carbidopa, and levodopa/benserazide), placebo or other treatments (including gabapentin, pregabalin, oxycodone–naloxone, and other drugs). Regarding drug types, we divided patients into subgroups according to whether they were treated with immediate-release drugs, transdermal application, or other type.

### Data Extraction

Using a unified form, 3 investigators independently extracted the data and created the data spreadsheet. Discrepancies were resolved via discussion. The extracted data mainly included study design, sample size (number of patients treated at least once with the trial drugs), age, proportion of male patients, duration of symptoms, duration of treatment, and number of augmentation events.

### Study Quality Assessment

Three investigators evaluated the quality of methodology of all included studies. The Cochrane Collaboration's tool for assessing bias (the Reviewer's Handbook^19^) was applied for randomized controlled trials, the Newcastle–Ottawa Scale (NOS)^20^ was used for cohort studies and open-label trials, the NOS^21^ was used for case–control studies, and the Agency for Healthcare Research and Quality (AHRQ)^22^ scale was used for cross-sectional studies and case series reports.

### Statistical Analysis

We investigated the augmentation rates for the total drug types and for the patient subgroups categorized by different treatment durations, interventions, drug types, and study design. In addition, we evaluated sensitivity and publication bias in the meta-analyses of the total drug types and subgroups. To investigate the incidence of augmentation with different treatment strategies as thoroughly as possible, for the interventional studies in which it was possible to split the intervention group and the control group, we used each group as an individual evaluation unit for statistical analyses. Prior to the meta-analysis of each item, Chi-squared tests were performed to test inter-trial heterogeneity; *P* ≥ 0.10 and I^2^ ≤ 40% indicated the absence of considerate heterogeneity, in which case a fixed-effects model (the inverse variance method) was applied; otherwise, a random-effects model (the Der-Simonian and Laird method) was applied. To compare other inter-trial differences, Chi-squared tests were used for enumeration data, and independent-samples *t* tests were used for measurement data. SPSS Predictive Analytics Software version 18.0 (SPSS, Inc., Chicago, IL), Meta-Analyst (Version Beta 3.13), and Comprehensive Meta-Analysis (Version 2) were used for the statistical analyses.

## RESULTS

### Search Results and Trial Characteristics

A total of 424 records were identified through searching of databases and other sources, and 60^8–11,13,16,23–76^ met the inclusion criteria. The included publications comprised 26 randomized controlled trials^9–11,13,23–44^ and 34 observational studies^8,16,45–76^(including 1 case–control study,^45^ 2 cohort studies,^46,47^ 13 cross-sectional studies or case series,^8,48–61^ and 18 open-label trials^16,62–76^). Of the included trials, 5^31,50,51,68,69^ were conducted in Asia, 30^9,16,23–26,30,32,34,36–38,41,43–46,54,57–60,62,63,66,71,73–76^ in Europe, and 15^8,11,27–29,47–49,53,55,56,61,64,65,72^ in North America. In addition, 10 trials^10,13,33,35,39,40,42,52,67,70^ involved intercontinental collaboration that included participants from Europe, North America, Australia, and/or Africa.

Based on the available data, 20 trials^8,9,13,23–28,31,32,34–36,39,49,57,58,69,76^ involved short-term treatment, and 37 trials^10,11,16,29,30,33,37,38,40–45,47,48,50–53,55,56,60–68,70–75^ involved long-term treatment; for 3 trials,^46,54,59^ the treatment duration was unclear (these 3 trials were not included in the analysis for short-term or long-term treatment). The number of RLS patients receiving long-term treatment accounted for 82.21% (2140/2603) of the total patients in 11 North American trials,^11,29,47,48,53,55,56,61,64,65,72^ 59.14% (3054/5164) of the total patients in 16 European trials,^16,30,37,38,41,43–45,60,62,63,66,71,73–75^ and 38.70% (416/1075) of the total patients in 3 Asian trials.^50,51,68^ The patients were treated with dopamine agonists in 40 trials,^10,13,23,27–30,33–43,45,47,48,52,53,55,57,58,61–63,65–74,76^ levodopa in 8 trials,^8,16,23,24,26,37,48,75^ gabapentin in 6 trials,^11,25,31,32,50,64^ pregabalin in 2 trials,^9,10^ combined medications in 9 trials,^37,46,48,49,51,54,56,59,60^ and oxycodone–naloxone in 1 trial.^44^ Transdermal application were used in 7 trials.^29,35,38,58,63,70,71^ Fifty-four trials^8–10,16,23–32,34–40,42–44,46–63,65–76^ reported augmentation events (529 patients), 2 trials^13,33^ reported hyperkinesia events (8 patients), 1 trial^11^ reported relapse events (31 patients), and 1 trial^64^ reported reemergence (0 patients). Eleven trials reported that the treatment medications had to be changed or discontinued in a portion of patients as a result of the above-mentioned events.^8,11,13,16,33,40,49,55,58,71,75^

The 60 analyzed studies included 11,543 patients; of these, 97.25% had a mean age of 56.28 ± 12.51 years, male patients accounted for 37.58% of 93.40% of the patients, 73.51% of the patients had a mean symptom duration of 11.57 ± 12.09 years, and 93.61% of the patients had a mean treatment duration of 65.65 ± 102.28 wk/person (median: 36). The overall article screening process is presented in Supplemental Figure 1, and the characteristics of the 60 included trials are presented in Table 1 .

**TABLE 1:**
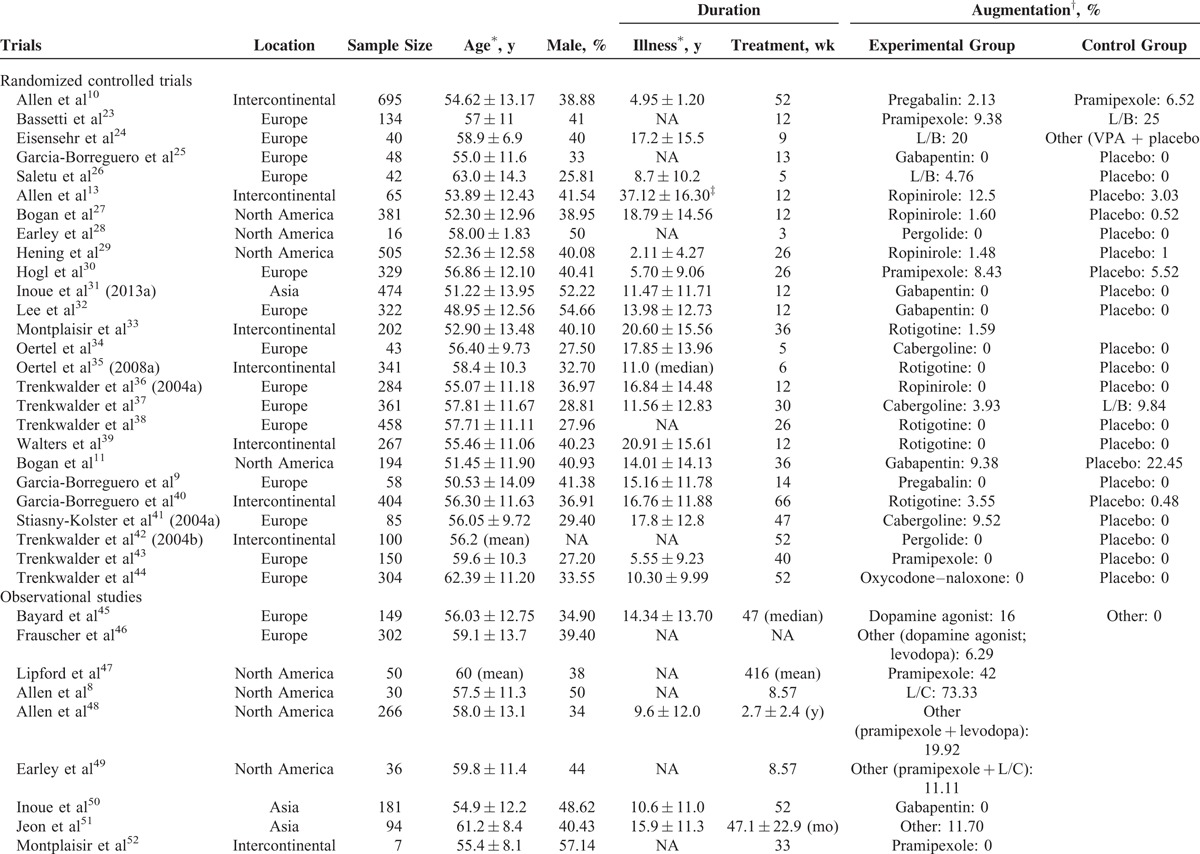
General Characteristics of the 60 Included Trials

**TABLE 1 (Continued):**
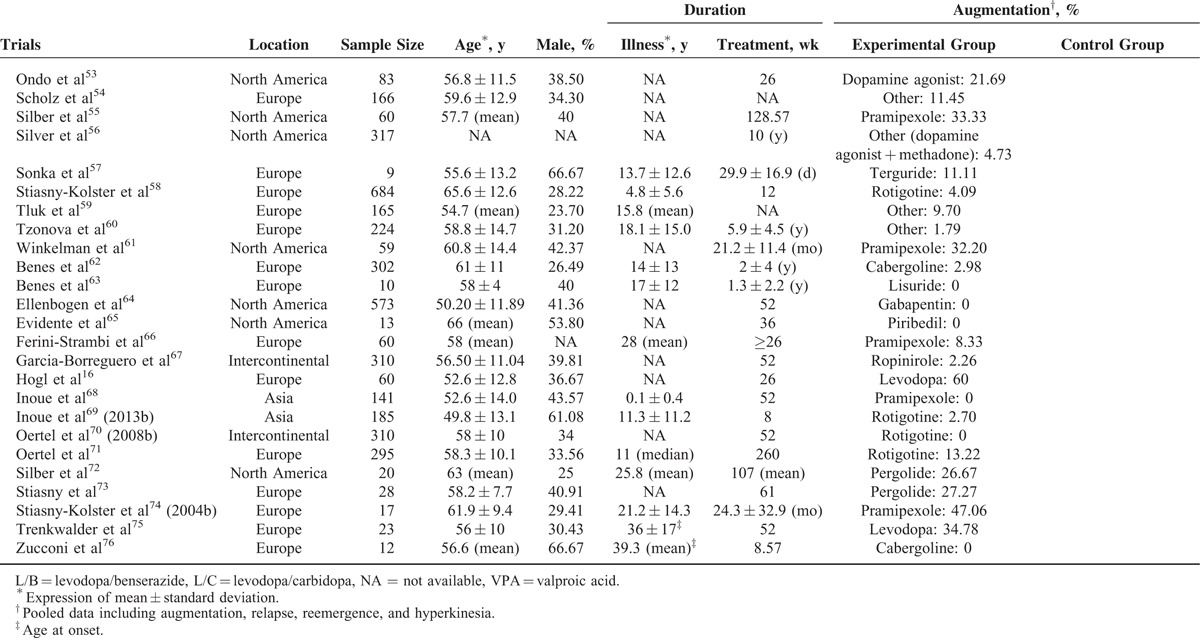
General Characteristics of the 60 Included Trials

### Quality Assessments

The quality assessments for cohort studies, case–control studies, and open label clinical trials are summarized in Supplemental Table 1. The quality assessments for case series and cross-sectional studies are summarized in Supplemental Table 2. With regard to RCTs, the individualized and overall quality assessments are demonstrated in Supplemental Figures 2 and 3, respectively. Overall, some of the included studies contain certain risks of bias for the study quality.

### Incidence Rates

**FIGURE 1:**
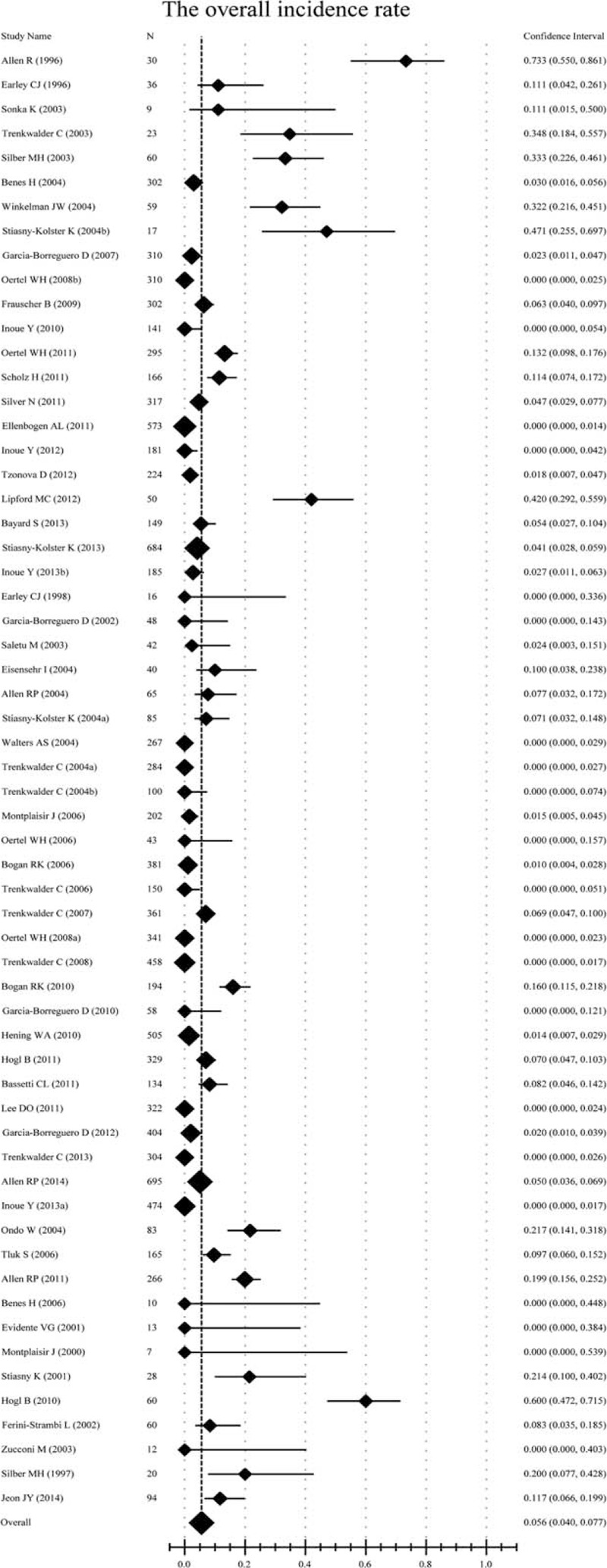
Forest plot of the incidence rate for the total drug types. The overall incidence rate was 5.6% (95% CI, 4.0–7.7); a random-effects model. CI = confidence interval.

The augmentation rate was 5.6% (95% confidence intervals (CI), 4.0–7.7; Table 2 and Figure 1) among all of the involved patients and drug types. Specifically, the augmentation rate was 6.1% (95% CI, 4.1–9.1) for patients undergoing long-term treatment, 3.3% (95% CI, 1.4–7.3) for patients undergoing short-term treatment, 27.1% (95% CI, 12.3–49.5) for patients taking levodopa, 6.0% (95% CI, 4.1–8.8) for patients taking dopamine agonists, 0.9% (95% CI, 0.2–3.3) for patients taking either pregabalin or gabapentin, 7.2% (95% CI, 5.0–10.3) for patients taking immediate-release drugs, and 1.7% (95% CI, 0.6–5.0) for patients taking transdermal application (Table 2 and Supplemental Figures 4–10). With regard to the geographic location, the incidence rate was 12.2% (95% CI, 6.6–21.4) in North America, 6.3% (95% CI, 4.1–9.4) in Europe, and 1.3% (95% CI, 0.2–6.2) in Asia (Table 2 and Supplemental Figures 11–13). With regard to the study design, the incidence rates were 2.3% (95% CI, 1.4–3.6) for randomized controlled trials, and 10.2% (95% CI, 6.8–15.1) for observational studies (Table 2 and Supplemental Figures 14 and 15).

**TABLE 2:**
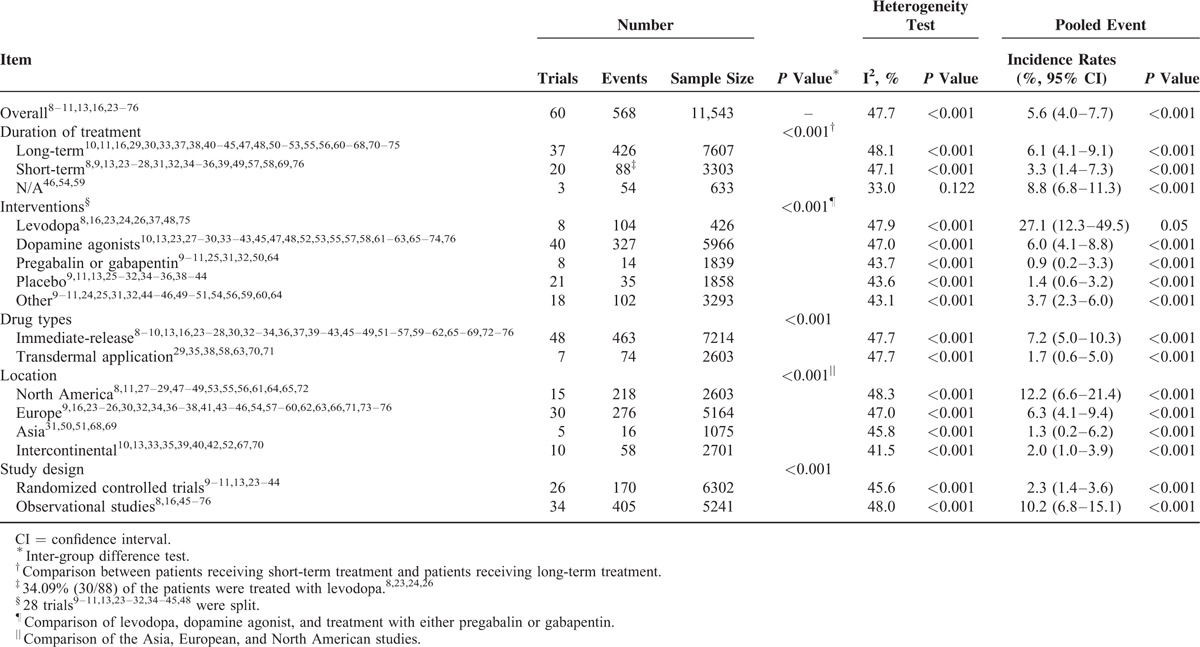
Statistical Results of the Incidence Rate

### Sensitivity Analysis

The sensitivity analysis indicated that the removal of any trial other than those by Allen et al^8^ and Hogl et al^16^ led to a *P* value >0.05 (Supplemental Table 3) when the Allen et al^8^ was included in the analysis, whereas the removal of any trial (including Hogl et al^16^) led to a *P* value <0.05 when the Allen et al^8^ was excluded for analysis, indicating that the meta-analysis results were affected by the Allen et al.^8^ For the meta-analyses of the total drug types and the 10 major subgroups, the removal of any trial (including Hogl et al^16^) led to a *P* value <0.001 (Supplemental Table 3), indicating that the results for these endpoints were robust. The removal of all observational studies or randomized controlled trials both led to a *P* value <0.001, indicating that the result was not affected by the study design.

### Publication Bias

**FIGURE 2:**
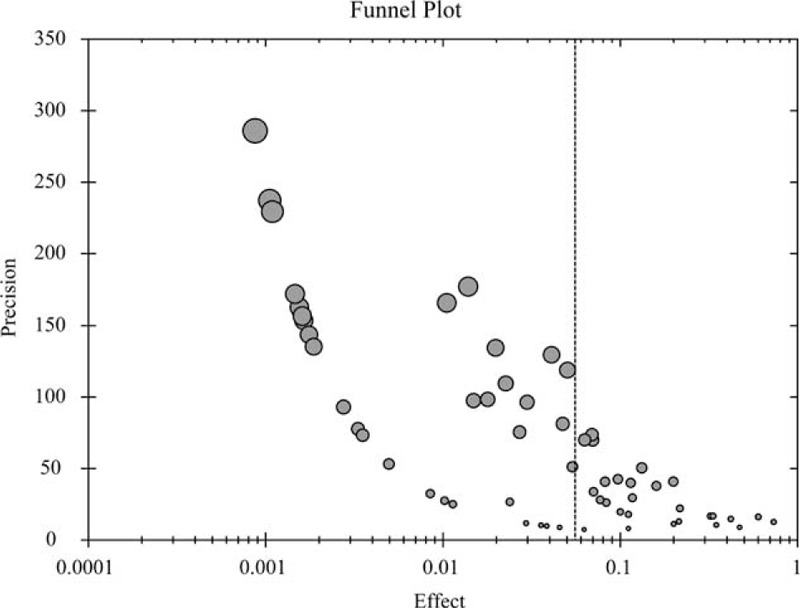
Funnel plot for the meta-analysis of the total drug types. The circles corresponding to the included trials were symmetrically distributes.

In the funnel plot for the total drug types, the circles corresponding to the included trials were symmetrically distributed (Figure 2). In addition, the fail-safe number was considerably higher than 60, and the *P* value of Begg test was >0.05 (Supplemental Table 4). These results indicated the absence of significant publication bias. For each of the 10 subgroups, the fail-safe number was higher than the number of trials included in the subgroup, and the *P* value of Begg test was >0.05 (Supplemental Table 4), indicating the absence of significant publication bias in the corresponding subgroup meta-analysis.

## DISCUSSION

This study demonstrated that the overall augmentation rate occurring during the treatment of primary RLS patients was 5.6% (95% CI, 4.0–7.7). Furthermore, the augmentation rate was higher in patients receiving long-term treatment than that in patients receiving short-term treatment. The rate was the highest for patients taking levodopa, followed by those taking dopamine agonists; the rate was the lowest in patients taking either pregabalin or gabapentin. The use of immediate-release drugs was associated with a higher incidence of augmentation compared with the use of transdermal application. The incidence was highest in North America, followed by Europe; the incidence was the lowest in Asia.

The following factors might contribute to the higher augmentation rate during long-term treatment compared with during short-term treatment: Downregulation of the density of dopamine receptors: Research has shown that the continuous use of type D2 dopamine agonists can result in a reduction of the number of dopamine D2 receptors.^77^ Some investigators have deduced that the long-term use of dopaminergic drugs may lead to augmentation through the downregulation of dopamine receptors.^78^ Reduced sensitivity of dopamine receptors: Research has revealed that the sensitivity of dopamine receptors decline in patients with long-term exposure to dopaminergic drugs.^78,79^ Overactivation of dopamine: It is suggested that because of an excessively high dopamine concentration in the central nervous systems of patients with augmentation,^79^ dopaminergic hyperstimulation^79^ and dopamine may overactivate spinal cord dopamine D1 receptors, leading to periodic leg movements.^79^

In this study, we found that the augmentation rate during short-term treatment reached 3.3% (95% CI, 1.4–7.3) and nearly a half of the cases were associated with the use of levodopa (Table 2). Hogl et al^16^ reported a median of 71 days of augmentation in a group of patients; considering the high medication doses that these patients used, the authors deduced that an over-accelerated increase in medication dosage might be an augmentation trigger.^16^ Other research also supported that high dosages likely increase the risk of augmentation.^80^ It is worth noting that, to confirm the ASRS, in Hogl et al^16^ relatively high dose of levodopa was used, and the rate of augmentation in this study is higher than others. However, the sensitivity analyses revealed that excluding this study does not significantly affect the overall findings in each analysis we performed.

Regarding the higher augmentation rate in patients taking levodopa compared with those taking other dopamine agonists, there are several potential explanations: Differences in the half-lives of the drugs. Compared with a drug with a shorter half-life, drugs with longer half-life reduce the risk of augmentation because they result in a decreased or eliminated pulsed stimulation of the dopamine receptors. Except for lisuride, dopamine agonists have significantly longer half-life than levodopa, thus may result in a lower augmentation rate. For example, pramipexole has a half-life of 8 to 12 h and results in an augmentation rate of 33% to 47%,^47,55,61,74^ and cabergoline has a half-life of 65 h and results in a low augmentation rate of 3%^62^; in contrast, levodopa has a half-life of 1 to 2 h and results in an augmentation rate as high as 60%^16^ to 73%.^8^ Furthermore, it is also thought that short-acting dopaminergic drugs, including levodopa, could lead to an augmentation rate that was higher than that of long-acting drugs.^1,7,12^ In this study, we found that the augmentation rate for transdermal application was remarkably lower than for immediate-release drugs, which indirectly supports the above hypothesis. Differences in the drugs’ ability to cross the blood–brain barrier (BBB). Compared with levodopa, dopamine agonists can directly cross the BBB and function in the brain without involving any transformation processes and without depending on the dopamine stored in the synaptic terminals. Differences in activity. Previous studies have revealed that, compared with the dopamine agonists, levodopa downregulates the density of dopamine receptors, causing a lower therapeutic efficacy. Differences in absorption. The difference of absorption between dopamine and levodopa might also be responsible for the higher augmentation rate in levodopa-treated patients. Levodopa is absorbed in the proximal small intestine, which is easily affected by food consumption, whereas the intestinal absorption of dopamine agonists does not necessarily compete with proteins or amino acids.

Augmentation was first discovered in patients taking dopaminergic drugs^8,53,55,61,62,66,72,74^; later in patients treated with nondopaminergic drugs, such as pregabalin^10^ and gabapentin^11^; furthermore there were examples of symptom deterioration in the placebo-treated group that met the criteria for augmentation.^12^ Our study also showed that the incidence rate of augmentation in the placebo-treated group was not zero. Although this phenomenon seems to be difficult to understand from the perspective of treatment outcomes, it might merely result from the natural disease course; that is, symptoms will worsen if the disease is not effectively treated. Moreover, a “placebo effect” in RLS treatment has been generally accepted; thus, the deterioration of placebo-treated patients is theoretically possible.

The original purpose of transdermal application was to minimize the fluctuation of serum drug concentration and reduce the instability of symptoms^35^ and gastrointestinal side effects^35,44^ associated with an immediate drug release rather than to avoid augmentation. However, in this study, we found that the augmentation rate of patients taking transdermal application was lower than that of patients taking immediate-release drugs. It has been reported that percutaneous administration can achieve a continuous drug release within 24 h, which effectively keeps the serum drug concentration relatively stable.^35,79^ In particular, continuous dopaminergic stimulation can reduce the risk of augmentation caused by fluctuating serum drug concentrations^10^ in patients (especially those with instable symptoms^58^) who have obvious symptoms in the early morning and during the day. Some investigators have even speculated that it is likely that augmentation can be avoided through the percutaneous administration of dopamine agonists.^12^ Regarding transdermal application, the risk of augmentation is low because of reduced fluctuation in the serum drug concentration. In the study by Maestri et al,^7^ the use of extended-release dopamine agonists in place of immediate-release drugs alleviated augmentation, a finding that indirectly supports our conclusion.

In our study, we detected that the rate of augmentation for pregabalin or gabapentin was significantly lower than that for dopaminergic drugs. This might be attributed to the following factors: Pregabalin and gabapentin are excreted through the kidneys in their original form without being metabolized in the liver.^81^ Therefore, different from dopaminergic drugs that can be gradually metabolized by the liver and lose their pharmacological effects, these 2 drugs can exert their pharmacological effects consistently until they are excreted from the body. In addition, for a portion of patients who suffer from multiple disease (eg, hypertension, diabetes, dyslipidemia) and have to take multiple drugs simultaneously, metabolism of dopaminergic drugs might be accelerated by some drugs that have an inductive effect on hepatic enzymatic activities (eg, simvastatin, atorvastatin). Nevertheless, gabapentin and pregabalin are not subject to the effect of hepatic enzyme inducers due to the fact that they are not metabolized in the liver. Pregabalin and gabapentin, the alpha-2-delta subunit of voltage-gated calcium channels,^82^ act as a presynaptic modulator of excitatory neurotransmitter release,^82^ can enhance slow-wave sleep,^25,83^ relieve pain,^25,83^ and improve sensory and motor symptoms in patients.^25^ Pharmacological effects of dopaminergic drugs are exerted partially through activation of dopamine D2 receptors. Dopamine D2 receptors mediate drug reward effects, through which their activation leads to a pleasure feeling, which can conceal or alleviate the discomfort with RLS symptoms in patients. However, repeated stimulation of dopamine D2 receptors can cause symptom rebounds, or symptom deterioration when the pharmacological effect weakens. The reward-seeking response of the body might be an important contributor to the fact that patients with augmentation often experience symptom occurrence or deterioration earlier in the day or at the time before the next drug administration, as well as the fact that patients taking dopaminergic drugs with a shorter half-life have a higher risk of augmentation. Pregabalin and gabapentin enacarbil are both extended-release agonists. Although up to date there are no clinical trials directly comparing short-acting drugs with long-acting agents, certain research supports that longer-acting agents may confer lower rates of augmentation.^10^

This study revealed that the augmentation rate was the highest in North America (12.2%), followed by that in Europe (6.3%), and it was the lowest in Asia (1.3%). Our statistical analyses demonstrated that the proportion of RLS patients receiving long-term treatment was also higher in North America than in Europe and the latter was higher than that of Asia. Therefore, selection bias might be a major contributor to the differences among the 3 regions, but the possibility of regional cultural and genetic backgrounds being influencing factors cannot be ruled out.

We want to acknowledge that, although in some included studies there was no detailed description for the methods of determining augmentation, authors of relevant studies did indicate that they did not detect augmentation. According to the guidelines of conducting meta-analysis of side effects, we included these studies in our assessment.

It is worth noting that in the current meta-analysis, there were heterogeneities between the included studies. These heterogeneities might be associated with differences in study design, intervention types, drug types, geographic locations, and treatment duration. However, another possible cause underlying these heterogeneities might be the lack of unified diagnostic criteria and available scoring systems in the early research. Although the MPI criteria and the ASRS^6^ were established and published in 2007, some trials did not use them and only provided symptom description. In addition, we cannot rule out the potential effect of variations across studies for collecting/evaluating endpoints, the duration of symptoms, previous medication history, and combined drug use.

Because of the insufficient data in the included studies, we could not evaluate the augmentation rates according to drug dosage, gender, and age and symptom severity. The participants in the 10 intercontinental collaborative trials^10,13,33,35,39,40,42,52,67,70^ came from Europe, North America, Australia, and Africa, and no specific data regarding the sample size and number of events in each region were available; therefore, we could not investigate the contribution of each study to the augmentation rate in each region. Because of the limited number of available studies, we did not evaluate differences in the augmentation rate between patients who were treated with different dopamine agonists and between drugs with different half-lives. Additionally, to determine the relative risks of augmentation with different drugs, the best approach can be to summarize all available evidence from randomized clinical trials and conduct network meta-analysis. We are conducting such a study for clarifying this. Despite the above limitations, this study successfully established the incidence rate of augmentation, with a hope to provide a clinical reference for the shared decision making of RLS treatment.

## CONCLUSIONS

Approximately 5 to 6 in 100 RLS patients develop augmentation during treatment. The augmentation rate during short-term treatment was lower than during long-term treatment, and patients taking either pregabalin or gabapentin were less likely to develop augmentation compared with those taking dopaminergic drugs. The augmentation rate for transdermal application was lower than that for immediate-release drugs.

## Supplementary Material

Supplemental Digital Content

## Supplementary Material

Supplemental Digital Content
