# Incidence of Augmentation in Primary Restless Legs Syndrome Patients May Not Be That High: Evidence From A Systematic Review and Meta-Analysis: Erratum

**Published:** 2016-02-12

In the article “Incidence of Augmentation in Primary Restless Legs Syndrome Patients May Not Be That High: Evidence From A Systematic Review and Meta-Analysis”,^1^ which appeared in Volume 95, Issue 2 of *Medicine*, the authors’ degrees were not presented correctly in the original article. The article has since been corrected online.

## References

[1] Liu GJ Wu L Wang SL Incidence of augmentation in primary restless legs syndrome patients may not be that high: Evidence from a systematic review and meta-analysis. *Medicine.* 2016 95:e2504. 26765466 10.1097/MD.0000000000002504 PMC4718292

