# Supplementary material for: Incidence of Augmentation in Primary Restless Legs Syndrome Patients May Not Be That High: Evidence From A Systematic Review and Meta-Analysis
Source: Medicine (Baltimore). 2016 Jan 15;95(2):e2504. doi: 10.1097/MD.0000000000002504 (PMC4718292; doi:10.1097/MD.0000000000002504)
Supplement: Supplemental Digital Content [file medi-95-e2504-s001.pdf]

Incidence of augmentation in primary restless leg syndrome patients may not be that high: evidence from a systematic review and meta-analysis

Search Terms and Databases

The search term was " (restless legs syndrome OR Willis-Ekbom disease) AND (dopaminergic agents OR levodopa OR levodopa/benserazide OR levodopa/carbidopa OR rr-L-dopa OR sr-L-dopa OR dopamine receptor agonist OR dopamine agonists OR pramipexole OR ropinirole OR pergolide OR rotigotine transdermal OR cabergoline OR terguride OR lisuride OR piribedil OR pregabalin OR gabapentin enacarbil OR gabapentin OR antiepileptic OR valproic acid OR methadone OR oxycodone-naloxone OR benzodiazepine OR opioids) AND (clinical trial OR clinical trials OR observational OR cross sectional OR longitudinal OR case control OR cohort OR open label OR case series) AND (augmentation OR rebound OR relapse OR malignant OR reemergence OR recurrence OR hyperkinesia)". The searched databases included PubMed, OVID, EMBASE, wiley citations, Web of Science research platform (including SciELO Citation Index, MEDLINE, KCI Korean Journal Database, the Web of ScienceTM Core Collection), and the Cochrane library

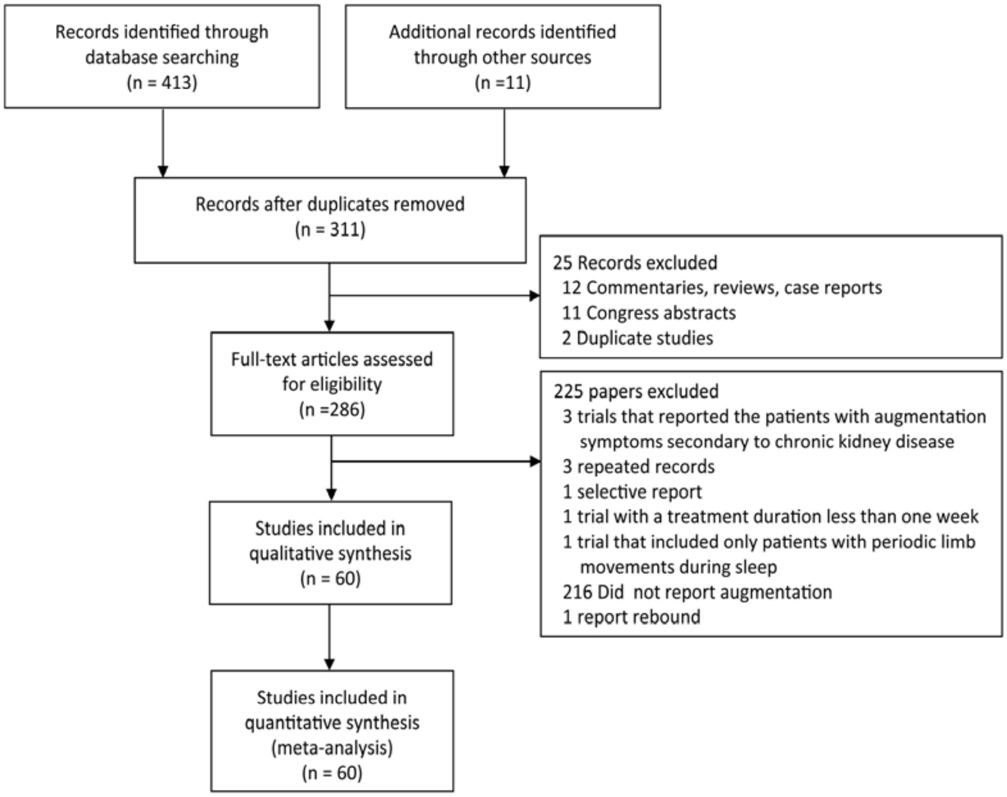

eFigure 1 Flow diagram of the screening process

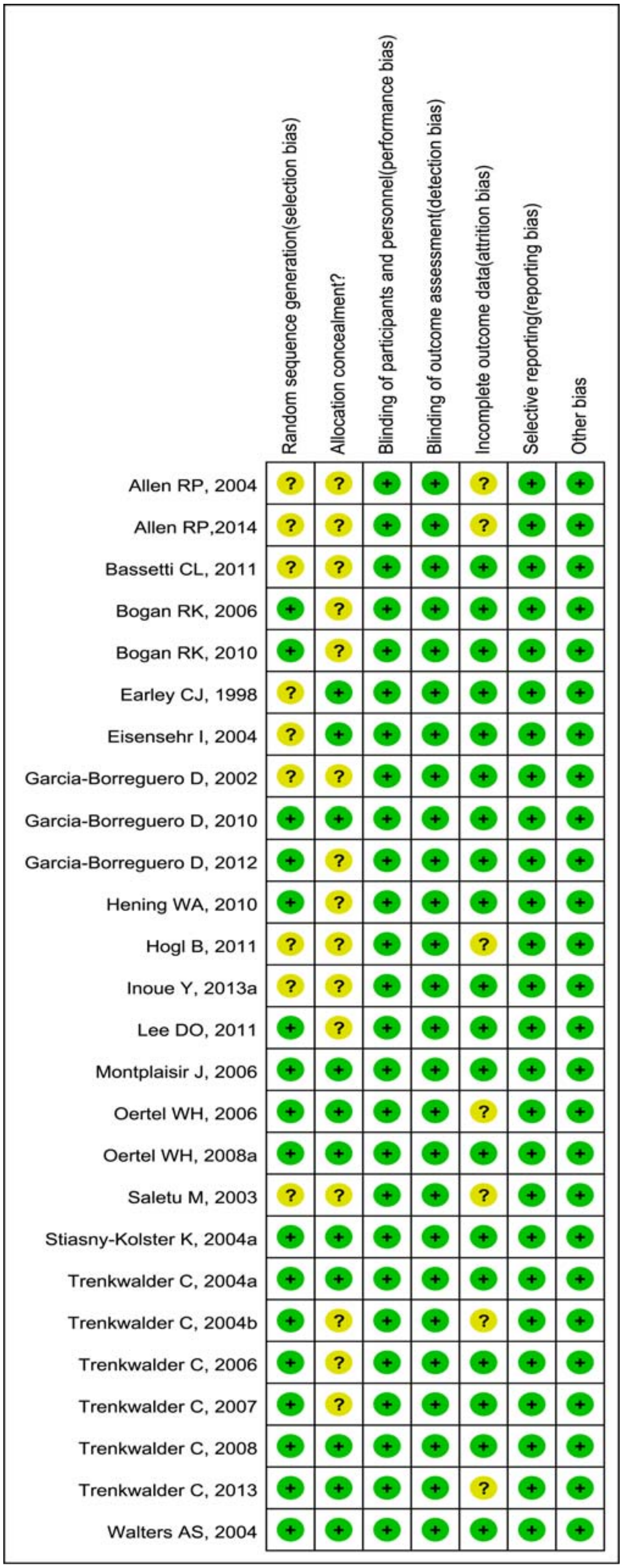

eFigure 2 Risk of bias graph for methodological quality

The results of the risk assessment of bias for all of the included trials are expressed as percentages.

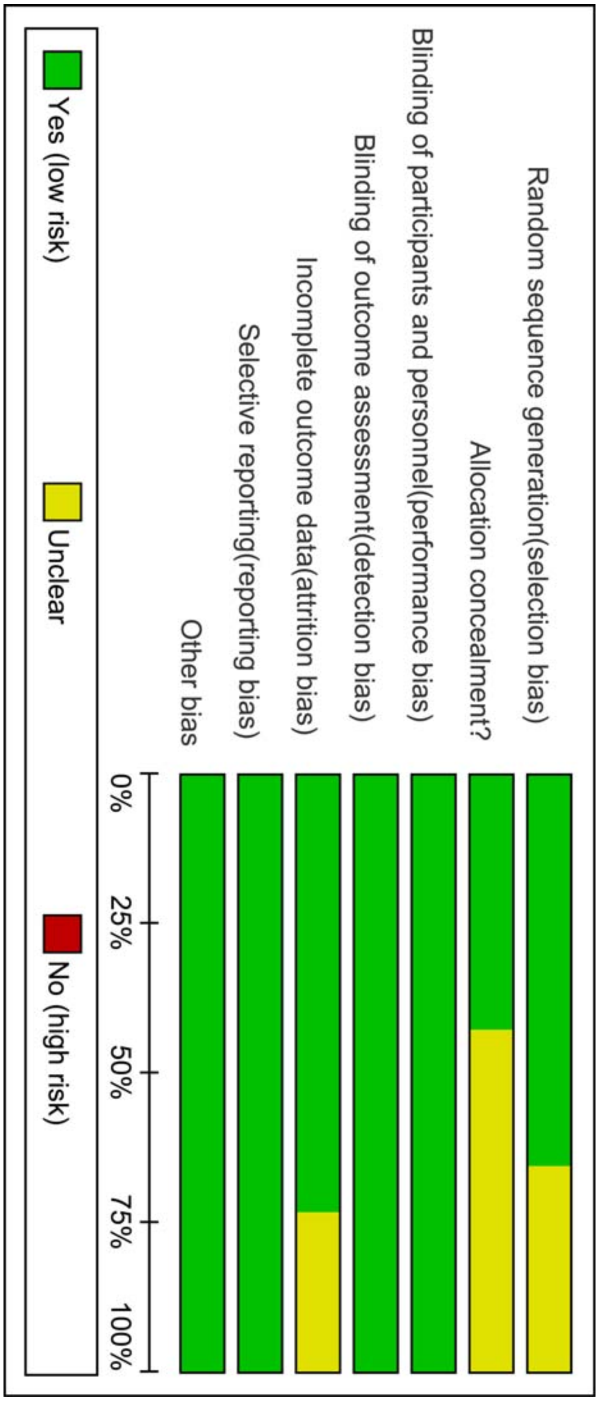

eFigure 3 Risk of bias summary for methodological quality

The result of the risk assessment of bias for each included trial is presented separately.

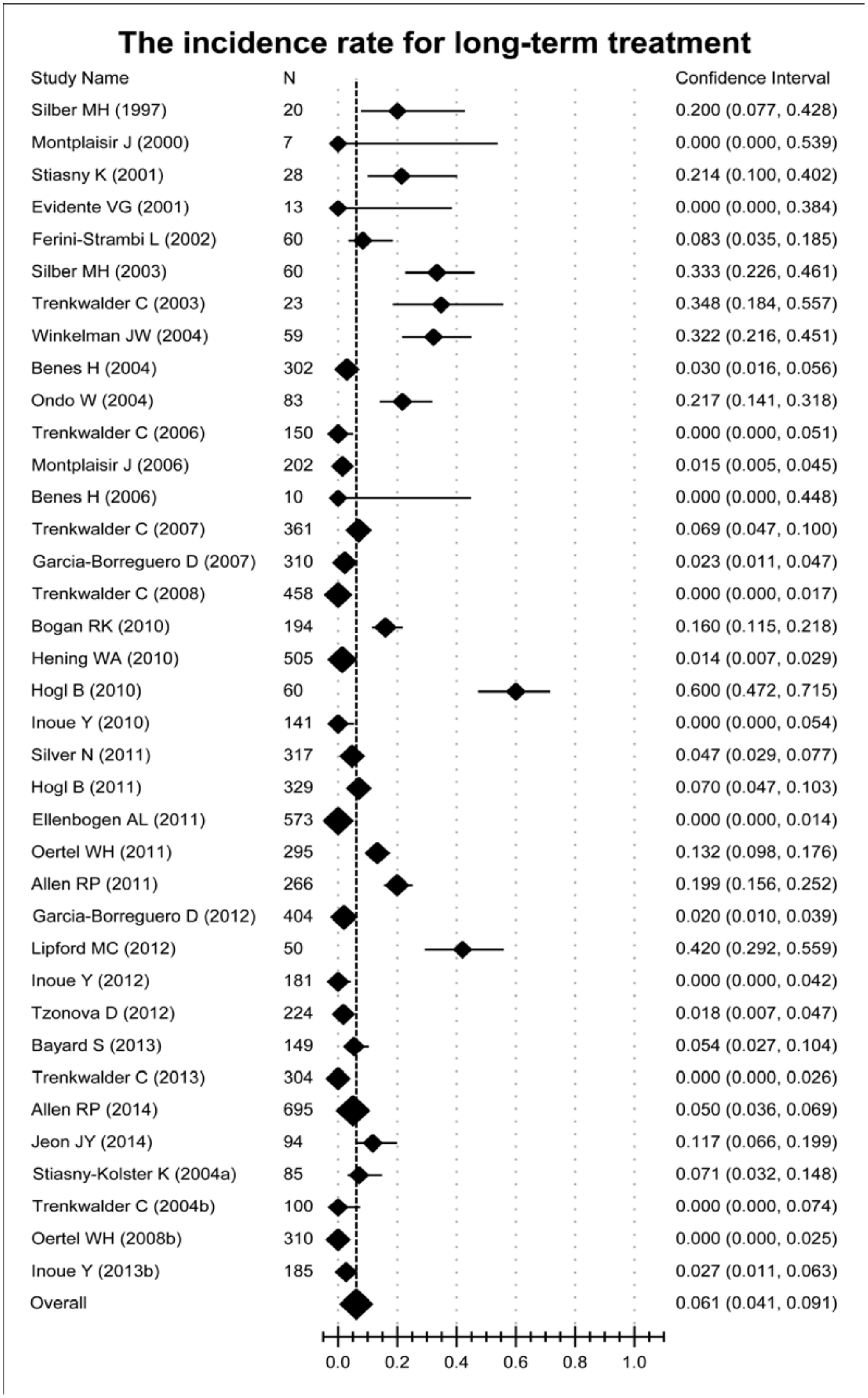

eFigure 4 Forest plot for the incidence rate of patients receiving long-term treatment

The incidence rate of patients receiving long-term treatment was 6.1% (95% CI, 4.1%-9.1%); a random-effects model was used.

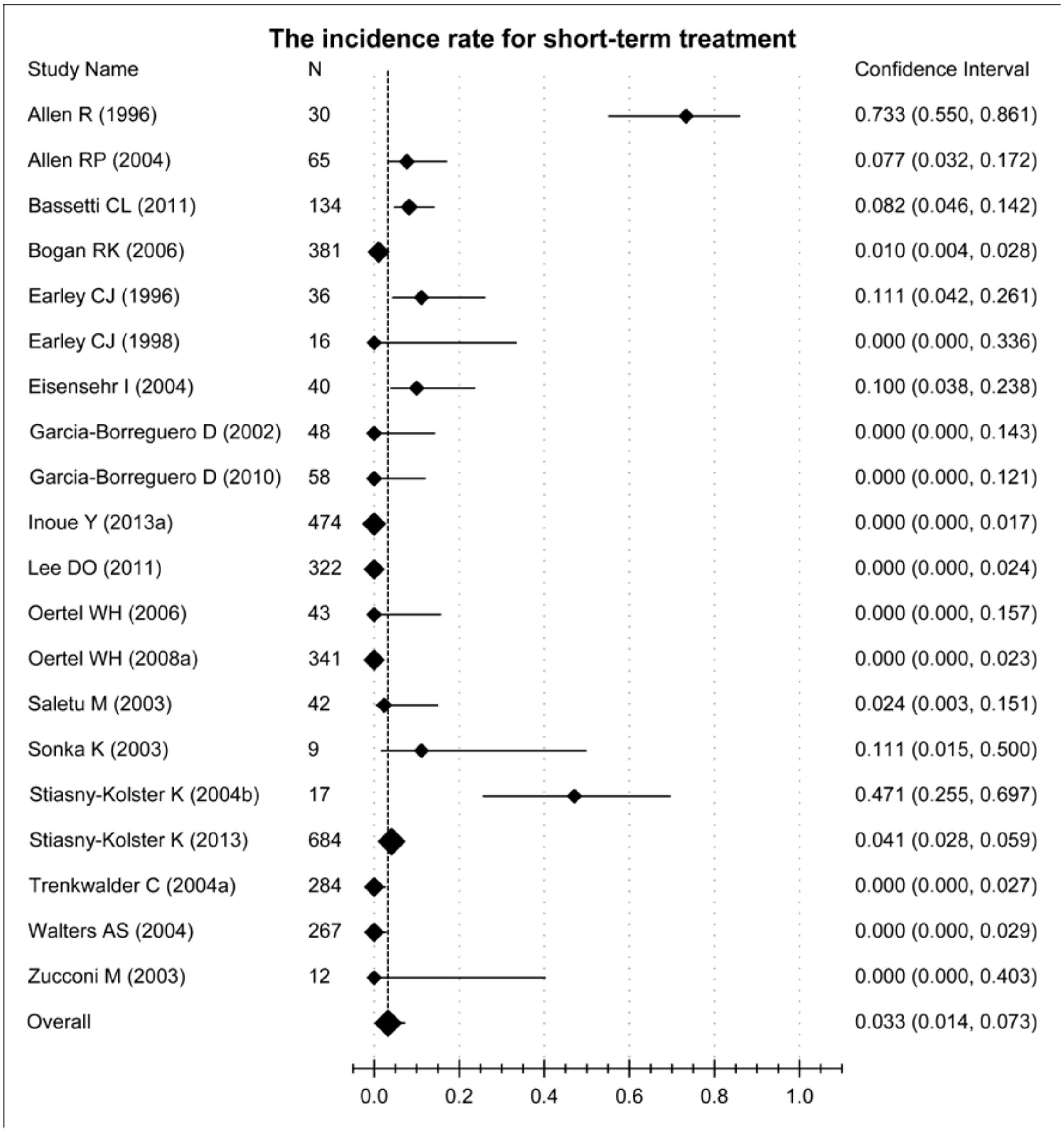

eFigure 5 Forest plot for the incidence rate of patients receiving short-term treatment

The incidence rate of patients receiving short-term treatment was 3.3% (95% CI, 1.4%-7.3%); a random-effects model was used.

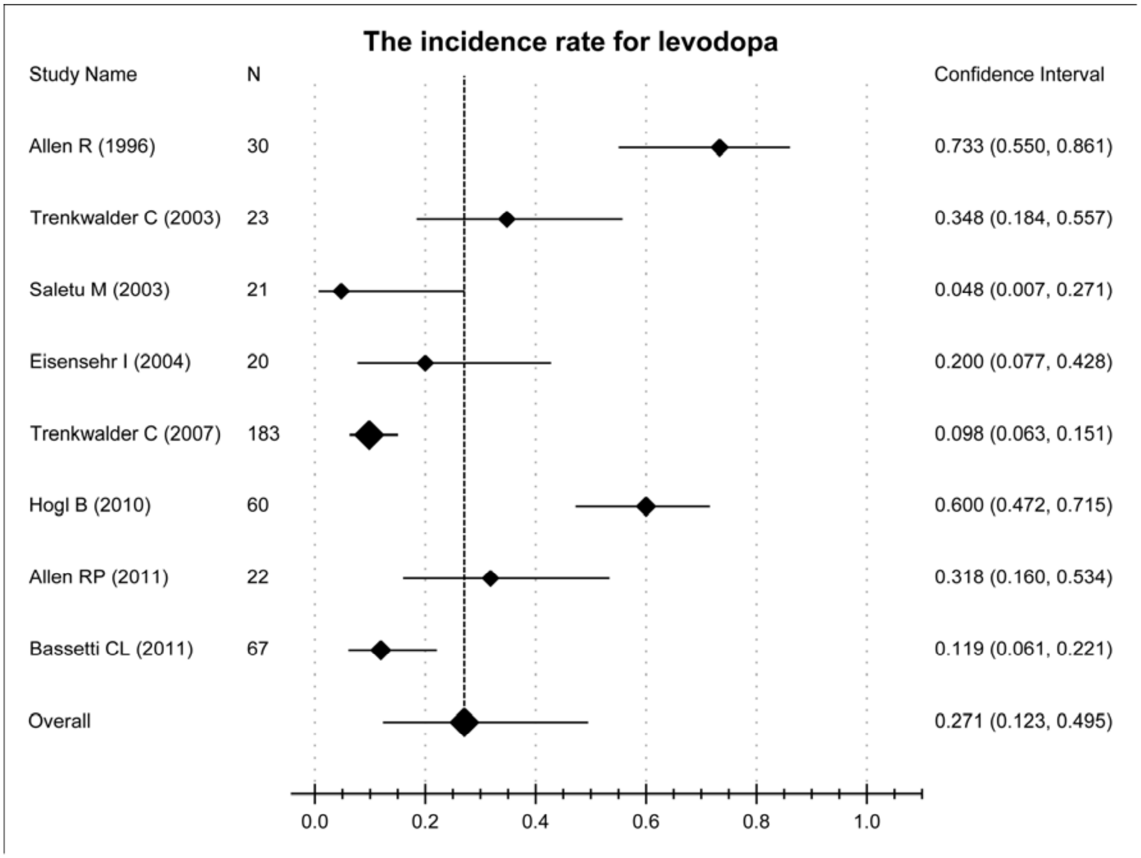

eFigure 6 Forest plot for the incidence rate of patients taking levodopa

The incidence rate of patients taking levodopa was 27.1% (95% CI, 12.3%-49.5%); a random-effects model was used.

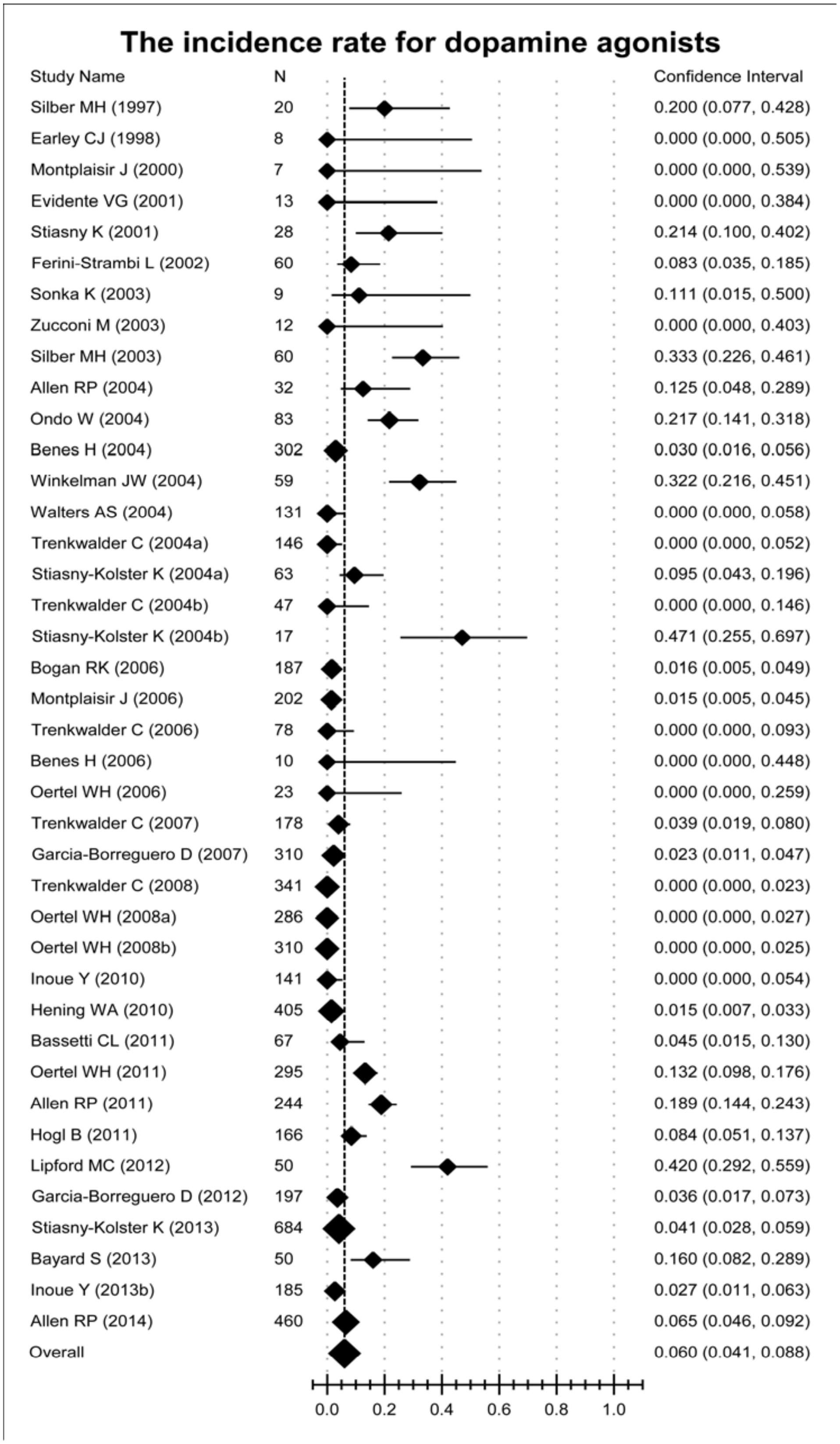

eFigure 7 Forest plot for the incidence rate of patients taking dopamine agonists

The incidence rate of patients taking dopamine agonists was 6.0% (95% CI, 4.1%-8.8%); a random-effects model was used.

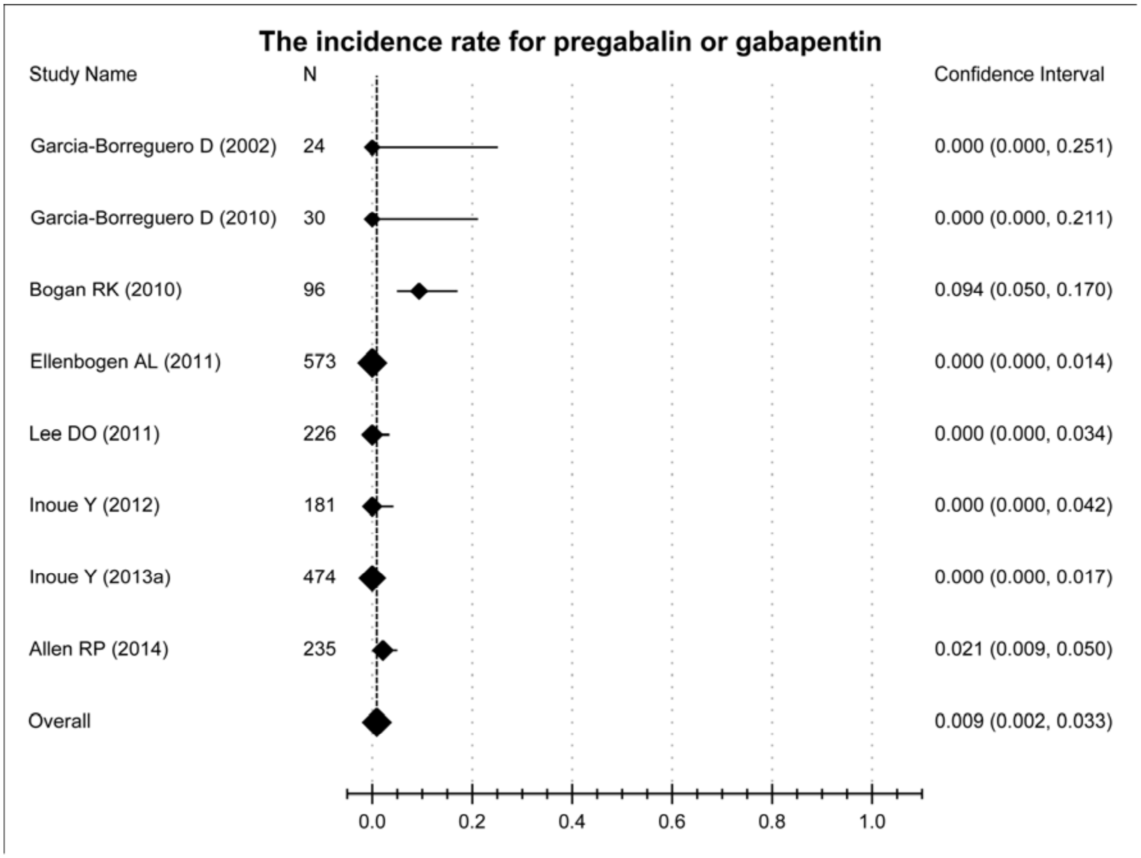

eFigure 8 Forest plot for the incidence rate of patients taking either pregabalin or gabapentin

The incidence rate of patients taking either pregabalin or gabapentin was 0.9% (95% CI, 0.2%-3.3%); a random-effects model was used.

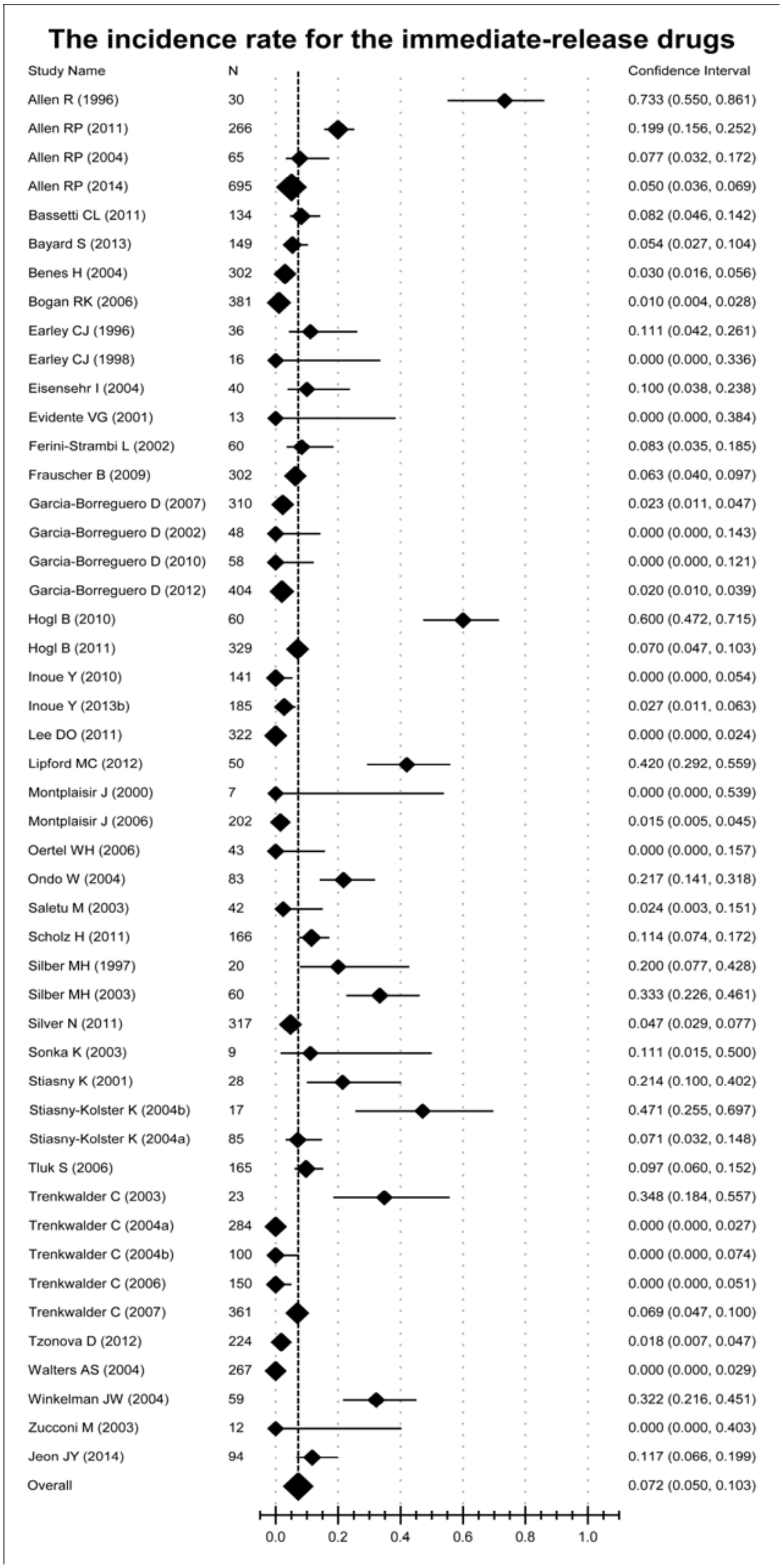

eFigure 9 Forest plot for the incidence rate of patients taking immediate-release drugs

The incidence rate of patients taking immediate-release drugs was 7.2% (95% CI, 5.0%-10.3%); a random-effects model was used.

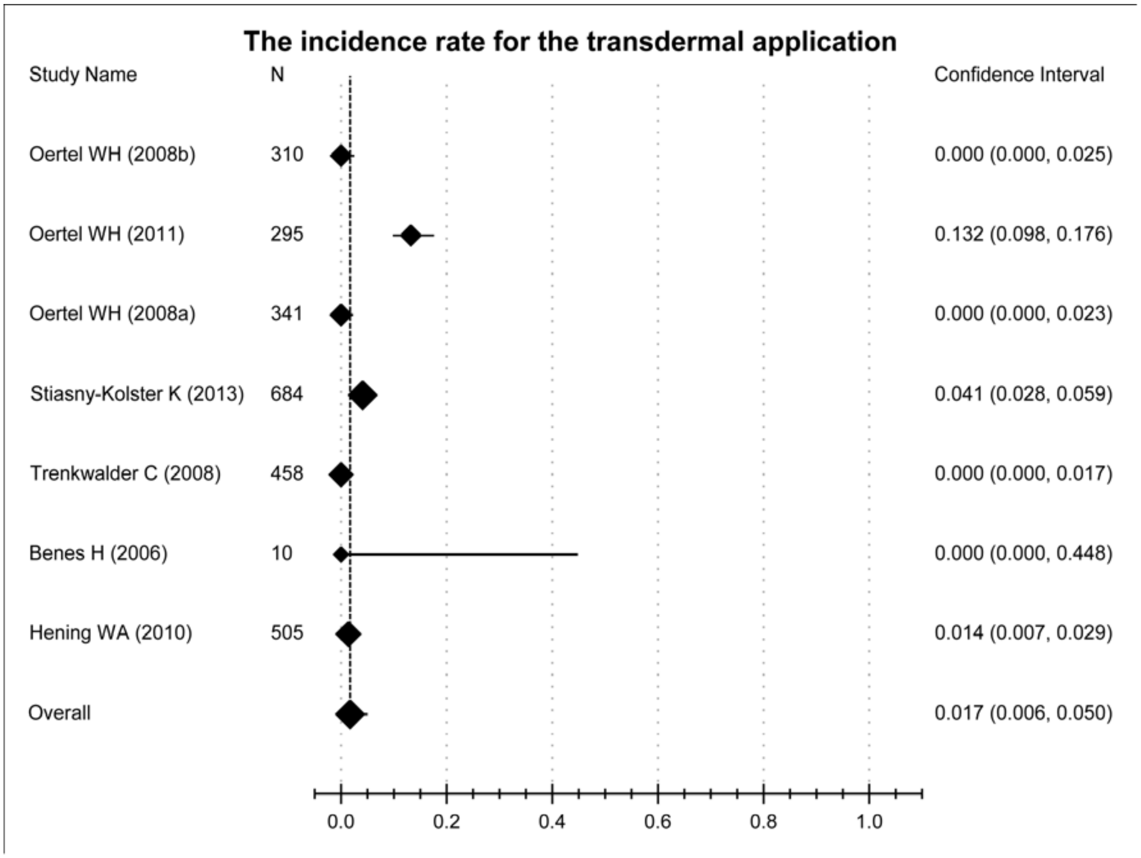

eFigure 10 Forest plot for the incidence rate of patients taking extended-release drugs

The incidence rate of patients taking extended-release drugs was 1.7% (95% CI, 0.6%-5.0%); a random-effects model was used.

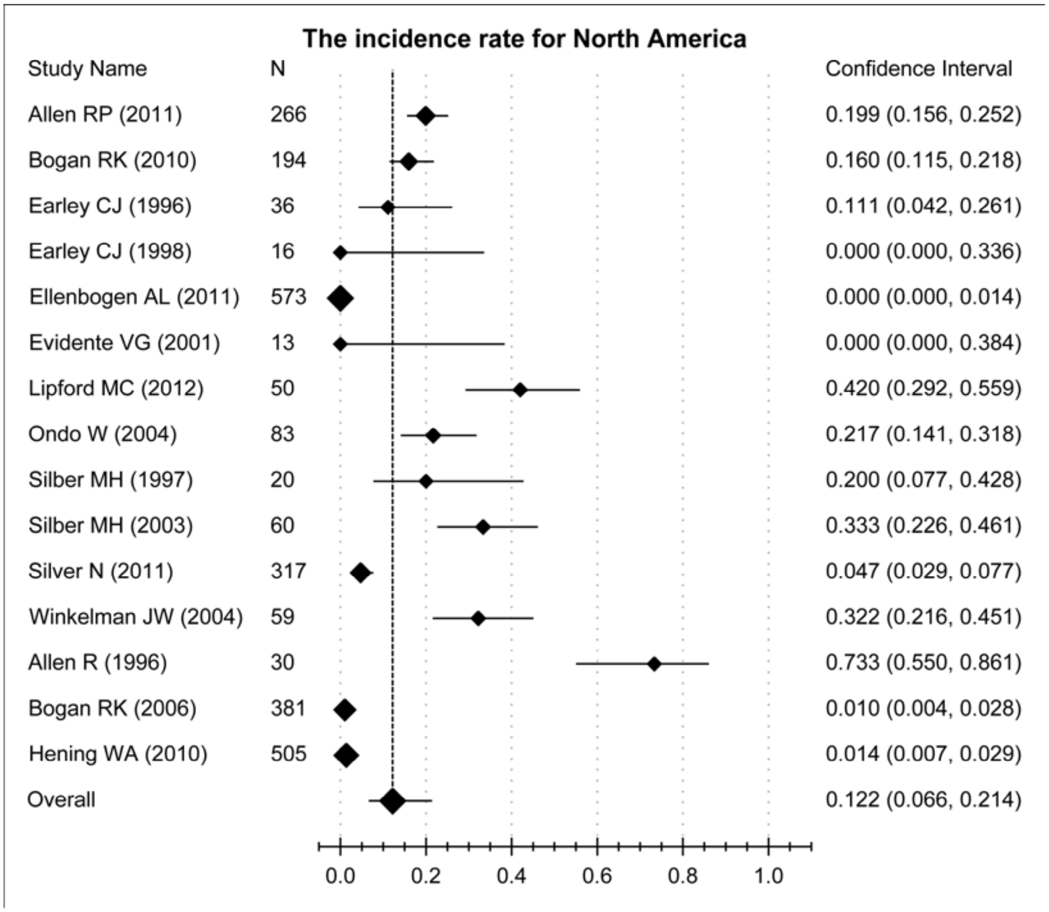

eFigure 11 Forest plot for the incidence rate of North America

The incidence rate of North America was 12.2% (95% CI, 6.6%-21.4%); a random-effects model was used.

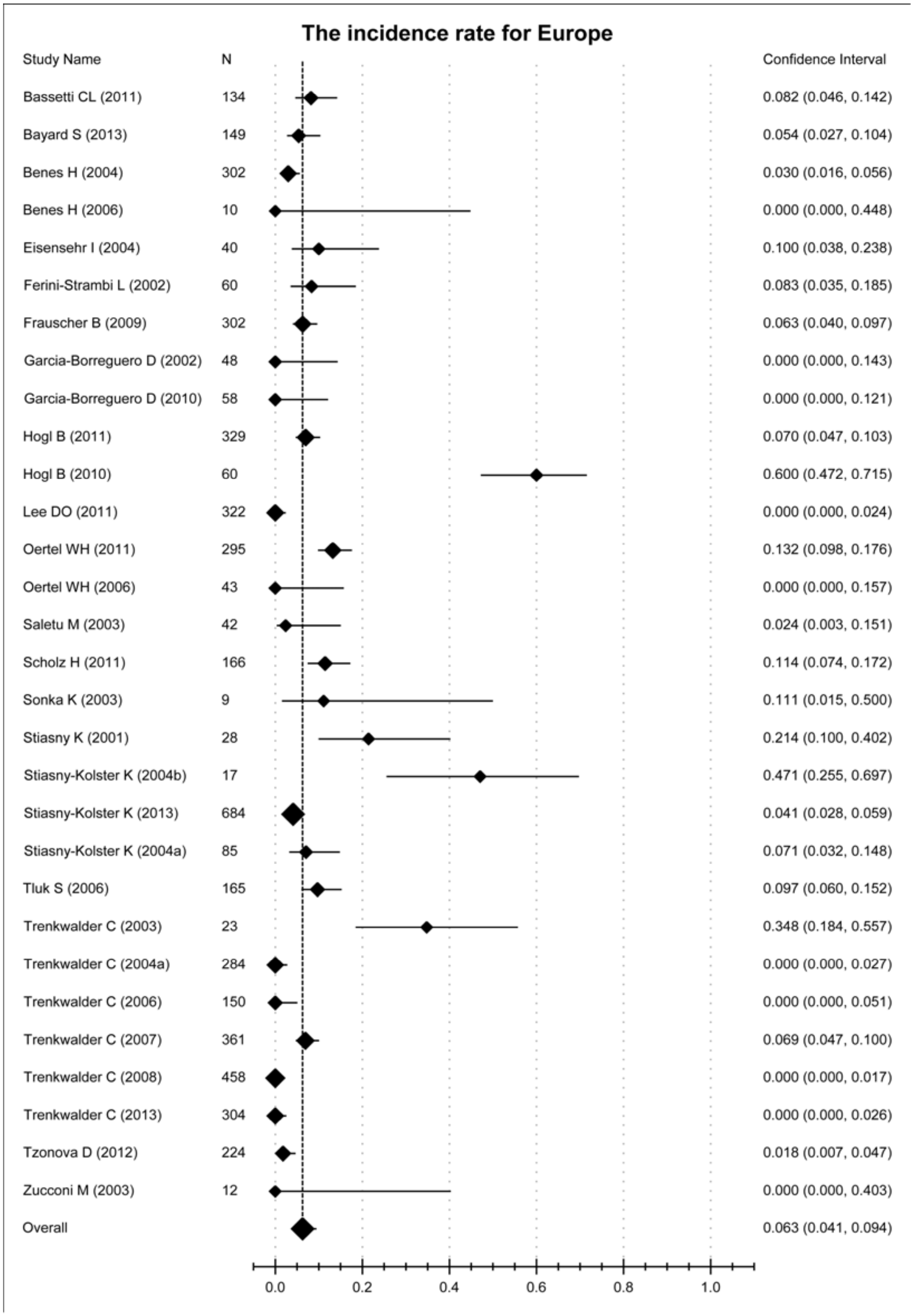

eFigure 12 Forest plot for the incidence rate of Europe

The incidence rate of Europe was 6.3% (95% CI, 4.1%-9.4%); a random-effects model was used.

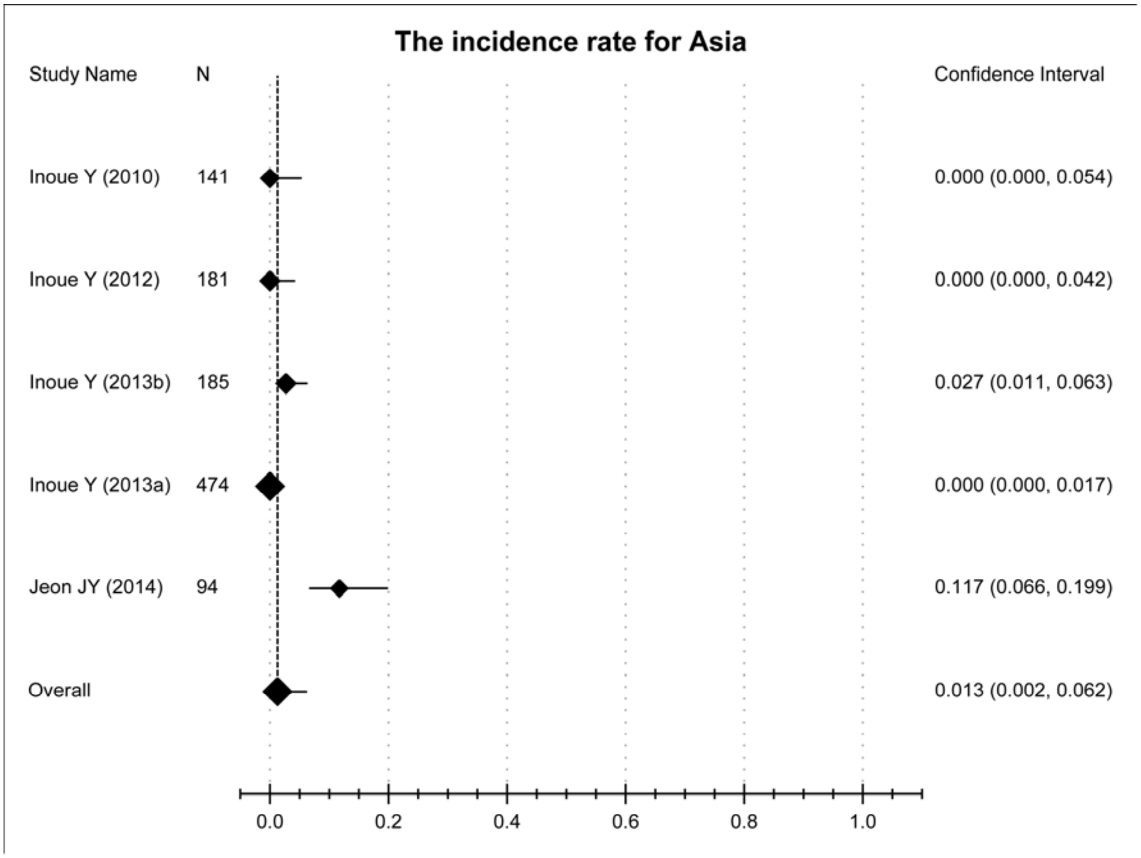

eFigure 13 Forest plot for the incidence rate of Asia

The incidence rate of Asia was 1.3% (95% CI, 0.2%-6.2%); a random-effects model was used.

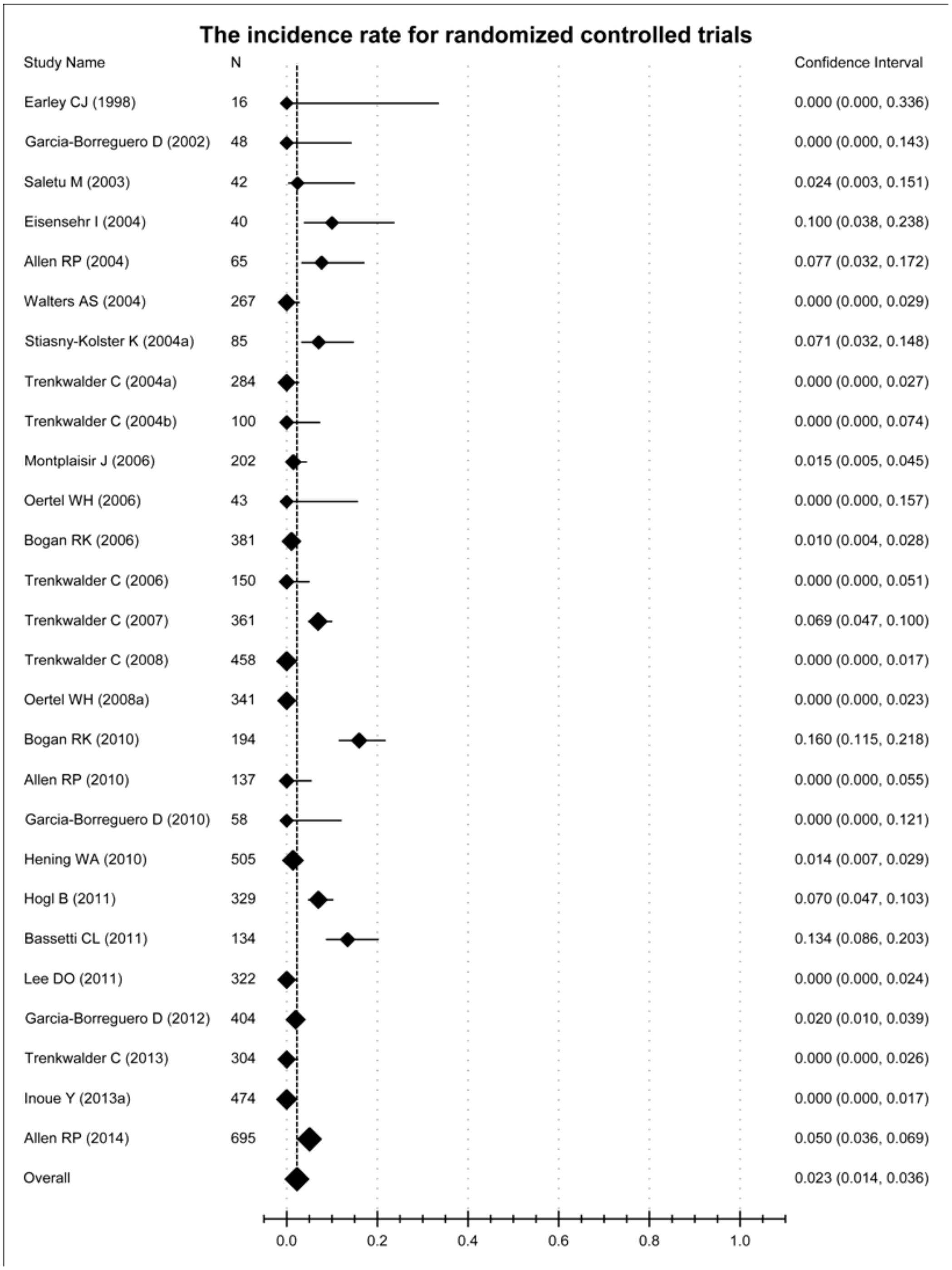

eFigure 14 Forest plot for the incidence rate of randomized controlled trials

The incidence rate of randomized controlled trials was 2.3% (95% CI, 1.4%-3.6%); a random-effects model was used.

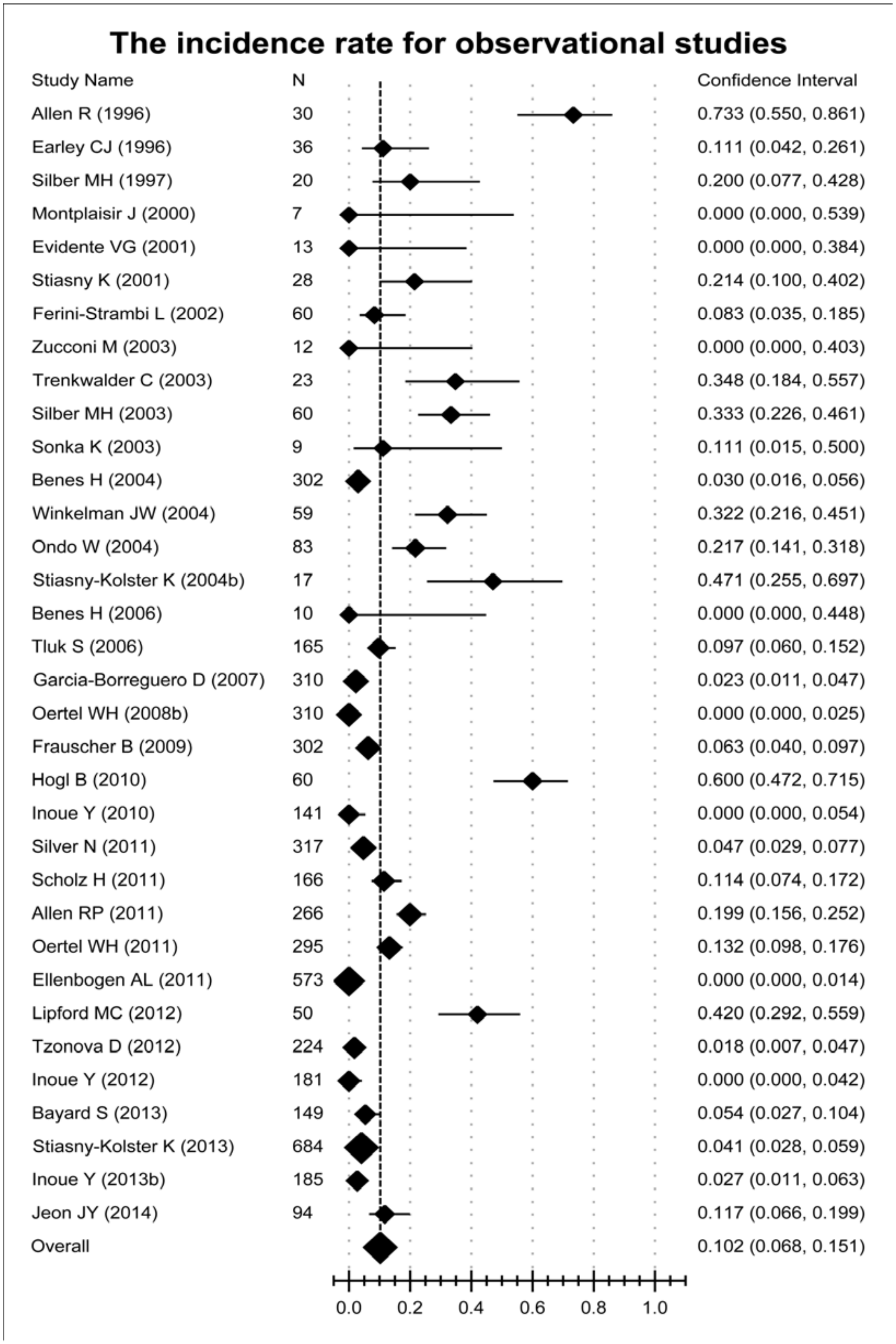

eFigure 15 Forest plot for the incidence rate of observational studies

The incidence rate of observational studies trials was 10.2% (95% CI, 6.8%-15.1%); a random-effects model was used.

eTable 1 Results of methodological quality evaluation for case-control studies, cohort studies and open-label trials

Trials	Selection (max 4*)	Comparability (max 2*)	Exposure (max 3*)
Bayard et al,[1] 2013	***	*	***
Frauscher et al,[2] 2009	***	**	**
Lipford et al,[3] 2012	****		***
Benes et al,[4] 2004	***		***
Benes et al,[5] 2006	***		**
Ellenbogen et al,[6] 2011	***	**	***
Evidente et al,[7] 2001	***		***
Ferini-Strambi et al,[8] 2002	***		***
Garcia-Borreguero et al,[9] 2007	****		***
Hogl et al,[10] 2010	****	**	***
Inoue et al,[11] 2010	***	**	***
Inoue et al,[12] 2012	***	**	**
Inoue et al,[13] 2013b	***		***
Oertel et al,[14] 2008b	***		**
Oertel et al,[15] 2011	***		***
Silber et al,[16] 1997	***		**
Stiasny et al,[17] 2001	***		**
Stiasny-Kolster et al,[18] 2004b	***		***
Stiasny-Kolster et al,[19] 2013	***		***
Trenkwalder et al,[20] 2003	***		**
Zucconi et al,[21] 2003	**	*	**

eTable 2 Results of methodological quality evaluation for cross-sectional studies and case series

Trials	Checklist item											Number		
	1	2	3	4	5	6	7	8	9	10	11	Yes	No	Unclear
Allen et al,[22] 1996	yes	yes	no	yes	yes	yes	yes	yes	unclear	yes	yes	9	1	1
Allen et al,[23] 2011	yes	yes	no	no	no	no	yes	yes	unclear	yes	yes	6	4	1
Earley et al,[24] 1996	yes	yes	no	yes	yes	yes	yes	yes	unclear	yes	yes	9	1	1
Jeozn et al,[25] 2014	yes	yes	yes	yes	no	yes	yes	yes	unclear	yes	yes	9	1	1
Montplaisir et al,[26] 2000	yes	yes	no	no	yes	no	yes	no	unclear	yes	yes	6	4	1
Ondo et al,[27] 2004	yes	yes	yes	yes	no	no	yes	yes	unclear	yes	yes	8	2	1
Scholz et al,[28] 2011	yes	no	yes	yes	no	no	yes	no	unclear	yes	yes	6	4	1
Silber et al,[29] 2003	yes	yes	yes	yes	no	no	yes	no	unclear	yes	yes	7	3	1
Silver et al,[30] 2011	yes	no	yes	yes	no	no	yes	no	unclear	yes	yes	6	4	1
Sonka et al,[31] 2003	yes	no	no	yes	no	no	yes	no	unclear	yes	yes	5	5	1
Tluk et al,[32] 2006	yes	no	no	yes	no	no	no	no	unclear	yes	yes	4	6	1
Tzonova et al,[33] 2012	yes	no	yes	yes	no	no	yes	no	unclear	yes	yes	6	4	1
Winkelman et al,[34] 2004	yes	yes	no	yes	no	no	yes	no	unclear	yes	yes	6	4	1

1=Define the source of information (survey, record review);

2=List inclusion and exclusion criteria for exposed and unexposed subjects (cases and controls) or refer to previous publications;

3=Indicate time period used for identifying patients;

4=Indicate whether subjects were consecutively included if the study was not population based;

5=Indicate whether the evaluators of subjective components of the study were masked to other aspects of the participants' status;

6=Describe any assessments undertaken for quality assurance purposes (e.g., test/retest of primary outcome measurements);

7=Explain any patient exclusions from analysis;

8=Describe how confounding factors were assessed and/or controlled;

9=If applicable, explain how missing data were handled in the analysis;

10=Summarize patient response rates and the completeness of data collection;

11=Clarify what follow-up, if any, was expected and the percentage of patients who had incomplete data or follow-ups.

eTable 3 Results after the removal of any trial

Item	Trials	Incidence rates (%)			p value
		Point estimate	Lower limit	Upper limit	
Overall	60	5.3-5.8	3.8-4.2	7.2-8.0	<0.001
Treatment duration					
Short-term	20	2.7-3.7	1.1-1.5	5.6-8.4	<0.001
Long-term	37	5.7-6.5	3.8-4.3	8.4-9.7	<0.001
Intervention					
Dopamine agonist	40	5.7-6.4	3.8-4.3	8.2-9.2	<0.001
Levodopa [a]	8	23.1-33.5	10.9-17.7	42.6-55.7	0.009-0.134
Levodopa [b]	7	18.6-27.5	8.7-13.9	29.9-48.4	<0.001-0.038
Pregabalin or gabapentin	8	0.7-1.3	0.1-0.3	1.9-4.5	<0.001
Drug types					
Transdermal application	7	1.0-2.3	0.2-0.8	3.2-6.8	<0.001
Immediate release	48	6.8-7.5	4.8-5.3	9.5-10.8	<0.001
Location					
Asia	5	0.7-2.2	0-0.5	3.2-11.4	<0.001
Europe	30	5.8-6.7	3.8-4.4	8.6-10.1	<0.001
North America	15	10.1-14.8	5.4-8.5	17.8-24.8	<0.001
Study design					
Randomized controlled trials	26	2.0-2.5	1.2-1.6	3.4-4.0	<0.001
Observational studies	34	9.4-11.0	6.3-7.3	13.8-16.1	<0.001

[a] Denotes the inclusion of the trial by Allen et al[22].

[b] Denotes the exclusion of the trial by Allen et al[22].

eTable 4 Results of the fail-safe numbers and Begg's test

Item	Number		p value (2-tailed) [a]
	Trials	Classic Fail-safe number	
Overall	60	10908	0.562
Treatment duration			
Short-term	20	1962	0.721
Long-term	37	3663	0.522
Intervention			
Dopamine agonist	40	2090	0.843
Levodopa	8	79	0.711
Pregabalin or gabapentin	8	386	0.536
Drug types			
Transdermal application	7	769	0.548
Immediate release	48	8006	0.588
Location			
Asia	5	188	0.462
Europe	30	7881	0.454
North America	15	1409	0.488
Study design			
Randomized controlled trials	26	7273	0.659
Observational studies	34	8165	0.553

[a] Kendall's tau with continuity correction.
