# Supplementary figures and images for: Incidence of Augmentation in Primary Restless Legs Syndrome Patients May Not Be That High: Evidence From A Systematic Review and Meta-Analysis
Source: Medicine (Baltimore). 2016 Jan 15;95(2):e2504. doi: 10.1097/MD.0000000000002504 (PMC4718292; doi:10.1097/MD.0000000000002504)

Figure that illustrate the control of hypertension in Myanmar

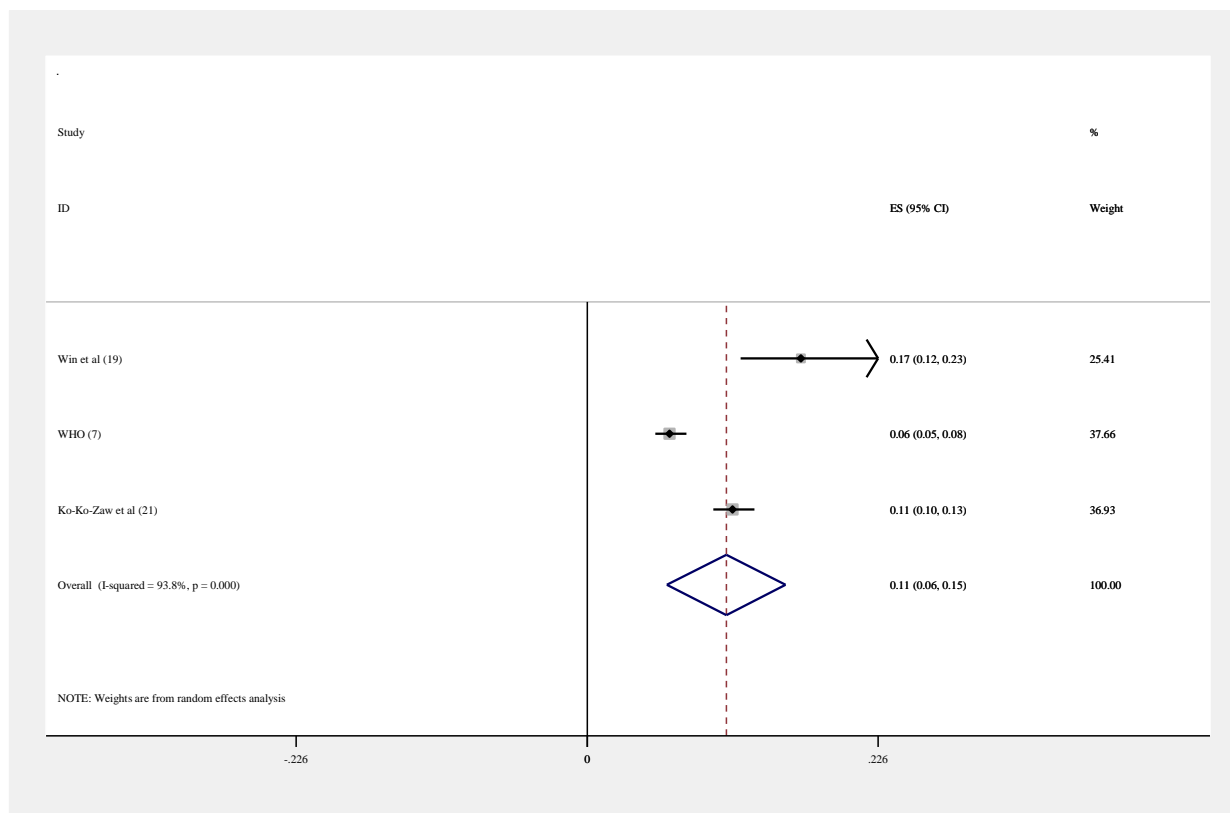

Supplement: Supplemental Digital Content [file medi-95-e2504-s002.pdf]
